# Molecular Events Involved in Influenza A Virus-Induced Cell Death

**DOI:** 10.3389/fmicb.2021.797789

**Published:** 2022-01-07

**Authors:** Rui Gui, Quanjiao Chen

**Affiliations:** ^1^CAS Key Laboratory of Special Pathogens and Biosafety, Center for Biosafety Mega-Science, CAS Center for Influenza Research and Early Warning, Wuhan Institute of Virology, Chinese Academy of Sciences, Wuhan, China; ^2^University of Chinese Academy of Sciences, Beijing, China

**Keywords:** cell death, influenza A virus, apoptosis, necroptosis, pyroptosis, viral infection

## Abstract

Viral infection usually leads to cell death. Moderate cell death is a protective innate immune response. By contrast, excessive, uncontrolled cell death causes tissue destruction, cytokine storm, or even host death. Thus, the struggle between the host and virus determines whether the host survives. Influenza A virus (IAV) infection in humans can lead to unbridled hyper-inflammatory reactions and cause serious illnesses and even death. A full understanding of the molecular mechanisms and regulatory networks through which IAVs induce cell death could facilitate the development of more effective antiviral treatments. In this review, we discuss current progress in research on cell death induced by IAV infection and evaluate the role of cell death in IAV replication and disease prognosis.

## Introduction

Apoptosis, necroptosis, and pyroptosis are the three main patterns of cell death in multicellular organisms, playing vital roles not only in embryonic development; normal tissue homeostasis; and diseases, such as atherosclerosis, cancer, and neurodegeneration, but also in defense reactions against infection (Bellamy et al., 1995; Galluzzi et al., 2008; Fuchs and Steller, 2011; Walsh, 2014; Jorgensen et al., 2017; Gao et al., 2018). The concept of apoptosis was proposed for the first time in 1972 (Kerr et al., 1972). Apoptosis can be activated by two distinct signaling pathways, namely, intrinsic (mitochondrial) and extrinsic (death receptor) pathways (Lowy, 2003). The term “necroptosis” was first used to describe necrotic cell death in 2005 (Degterev et al., 2005). In the next decade, several critical molecules, including receptor interacting protein kinase (RIPK)1/3 and mixed lineage kinase domain like pseudokinase (MLKL) were found to be involved in necroptosis (Holler et al., 2000; Degterev et al., 2005; Cho et al., 2009; He et al., 2009; Zhang et al., 2009; Murphy et al., 2013). Additionally, pyroptotic cell death was first observed in 1986, when a research team found that cultured primary mouse macrophages induced cell death and leakage of cell contents when treated with the lethal toxin anthrax (Friedlander, 1986). In subsequent decades, follow-up research showed that pyroptosis relies on a gasdermin family member to induce cell rupturing and release cellular contents (Kayagaki et al., 2015; Liu et al., 2016; Aglietti and Dueber, 2017; Shi et al., 2017). Although cross-talk exists between apoptosis, necroptosis, and pyroptosis, apoptosis has minimal impact on neighboring cells because it is a non-inflammatory type of cell death; this is the main difference between apoptosis, necroptosis, and pyroptosis. Moreover, in necroptosis and pyroptosis, the release of cellular contents is an intense trigger of innate and adaptive immune responses (Henson et al., 2001; van Delft et al., 2010; Wallach et al., 2016; Shi et al., 2017).

Influenza A virus (IAV) is a negative-sense single-stranded RNA virus that is a member of the Orthomyxoviridae family (Yamauchi, 2020). IAV is among the most notorious respiratory diseases, causing almost 3–5 million cases of critical illness and approximately 250,000–500,000 deaths worldwide each year (Walsh, 2014; Hartmann et al., 2017; Doyon-Plourde et al., 2019). IAV mainly infects the airway epithelial cells, leading to inflammation and cell death, followed by respiratory tract damage, pneumonia, and even respiratory dysfunction or failure (Le Goffic et al., 2007; Cardani et al., 2017; Atkin-Smith et al., 2018). Severe IAV infection is always accompanied by hypercytokinemia, also known as cytokine storm, which is closely related to disease severity and could be a predictor of disease progression and mortality (De Jong et al., 2006; Guo et al., 2015; Zhang Y. et al., 2020). However, cell death and inflammation do not always harm the host; moderate cell death and inflammation can facilitate the elimination of viruses (Kuriakose et al., 2016; Zheng et al., 2020). Therefore, exploring the relative contribution of cell death and inflammation to suppression of viral replication and improvement of prognosis vs. disruption of host homeostasis and physical function, is particularly important for developing novel treatment strategies (Brandes et al., 2013).

In this review, we discuss the mechanisms of the three types of cell death (apoptosis, necroptosis, and pyroptosis) during IAV infection to address how IAV triggers and manipulates cell death and how these processes determine the final fates of viruses, cells, and hosts.

## Apoptosis During Influenza a Virus Infection

Characteristic morphological features of intrinsic apoptosis, including flowing nuclear and cytoplasmic condensation, fragmentation of cellular DNA and membrane blebbing with apoptotic body formation during IAV infection were observed in previous studies (Takizawa et al., 1993; Fesq et al., 1994; Hinshaw et al., 1994; Mori et al., 1995). However, the specific mechanisms through which IAV induces apoptosis and the roles of apoptosis in IAV infection remain unclear. In recent years, apoptosis has been documented as a cell defense strategy against invading viruses in most multi-cellular organisms (Robert et al., 2012). Unfortunately, in the battle between viruses and their hosts, viruses have also evolved the ability to subvert apoptosis in the host cells (Danthi, 2016; Orning et al., 2018). Thus, apoptosis has both positive and negative effects on the life cycle of IAV (Galluzzi et al., 2008). In general, early apoptosis reduces viral replication, whereas late apoptosis promotes the release and dissemination of progeny viruses (Zhou et al., 2017).

## Intrinsic Apoptosis

IAV primarily induces intrinsic apoptosis through some viral proteins and the IAV progeny ribonucleoprotein complex (vRNP) (Table 1). Research on apoptosis has revealed details of the mechanisms underlying IAV-induced apoptosis (Cardani et al., 2017; Zhou et al., 2017; Atkin-Smith et al., 2018).

**TABLE 1:** Viral proteins of IAV that are involved in cell death.

IAV protein	Subtypes	Molecular mechanism	Types of death
HA	H1N1	- HA promotes apoptosis by enhancing host cell endoplasmic reticulum stress (Frabutt et al., 2018). - Unknown (Hartmann et al., 2017).	Apoptosis Necroptosis
NA	H1N1 H3N2	- NA stimulates endoplasmic reticulum stress, enhancing apoptosis (Slaine et al., 2021). -NA interacts with CEACAM6 protein, which increases the tyrosine phosphorylation of FAK, AKT, GSK3β, and Bcl-2, and inhibits cell death (Gaur et al., 2012).	Apoptosis
NP	H1N1 H1N1 H3N2	- NP directly interacts with the anti-apoptotic protein CLU, which inhibits apoptosis by interacting with Bax, thereby interfering with the function of CLU (Tripathi et al., 2013). - NP promotes apoptosis by down-regulating API5 expression; this interferes with E2F1 recruitment to the *Apaf-1* promoter, leading to down-regulation of *Apaf-1* and impairment of apoptosome formation (Morris et al., 2006; Mayank et al., 2015). - NP interacts with RNF43, which can inhibit apoptosis by interacting with Bax and impairing RNF43 function (Nailwal et al., 2015).	Apoptosis
NS1	H5N1 H1N1	- Unknown (Schultz-Cherry et al., 2001; Lam et al., 2008). - NS1 interacts with MLKL to induce oligomerization and membrane translocation (Gaba et al., 2019).	Apoptosis Necroptosis
NS2	-	-	-
M1	H1N1	- M1 directly interacts with Hsp70, which then interacts with *Apaf-1* to disrupt apoptosome formation (Halder et al., 2011).	Apoptosis
M2	H1N1	- M2 interacts with the ATG5/Beclin-1 complex to inhibit autophagosome fusion, promoting apoptosis (Gannage et al., 2009). - M2-mediated perturbation of ion concentrations triggers activation of the NLRP3 inflammasome (Ichinohe et al., 2010).	Apoptosis Pyroptosis
PB1-F2	H3N2 H1NI H1N1	- PB1-F2 activates the NLRP3–ASC inflammasome (McAuley et al., 2013). - PB1-F2 localizes to the mitochondria and alters mitochondrial permeability (Gibbs et al., 2003).	Pyroptosis Apoptosis
PA-X	-	-	-
PA	H1N1	- vRNPs are sensed by DAI/ZBP1, which then triggers the downstream cell death signaling pathway (Kuriakose et al., 2016; Thapa et al., 2016).	Apoptosis
PB1			Necroptosis
PB2			Pyroptosis

These mechanisms may be classified into three types based on how viral proteins of IAV activate apoptosis. First, viral proteins interact with a host protein. Subsequently, mitochondrial membrane permeability is increased, which activates other specific signaling pathways to induce apoptosis (Chen et al., 2001). Second, vRNPs interact directly with DNA-dependent activator of interferon (IFN)-regulatory factor (DAI), which activates caspase-8 and then caspase-3, to induce apoptosis (Thapa et al., 2016). Third, upon IAV infection, endoplasmic reticulum (ER) stress and autophagy are triggered, thereby inducing apoptosis (Frabutt et al., 2018; Yeganeh et al., 2018). Fourth, some viral proteins can regulate apoptosis directly; however, the details of this mechanism are still unclear (Schultz-Cherry et al., 2001).

**FIGURE 1:**
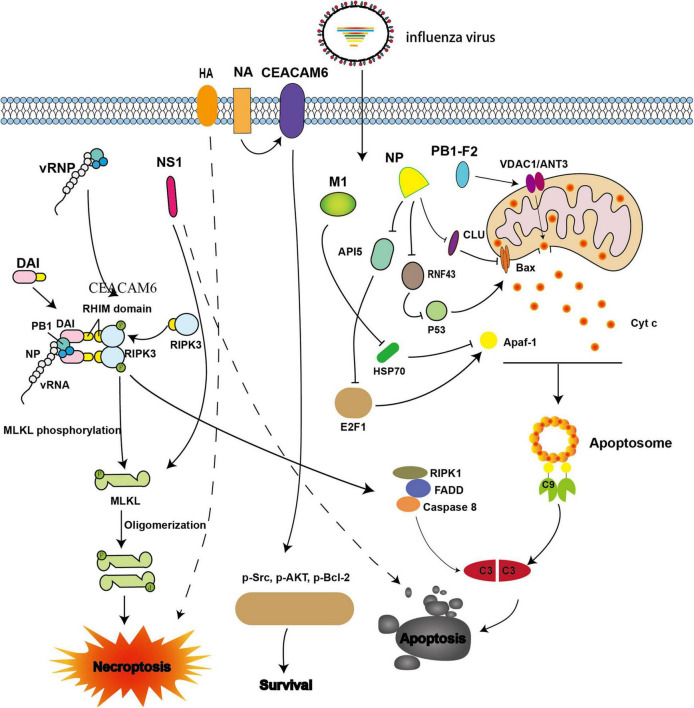
Viral proteins involved in intrinsic apoptosis and necroptosis during influenza A virus (IAV) infection. The IAV progeny ribonucleoprotein complex (vRNP) is sensed by DAI via recruitment of RIPK3 to activate MLKL. NS1 directly interacts with MLKL and induces its oligomerization. NS1 also can induce apoptosis; however, the underlying mechanism is not well understood. NA interacts with CEACAM6 and increases the phosphorylation of Src, AKT, and Bcl-2, thereby inhibiting apoptosis. When M1 binds with Hsp70, apoptosome formation is blocked. When NP interacts with CLU and RNF43, its ability to resist apoptosis is weakened by interference with the mitochondrial translocation of Bax. NP also promotes apoptosis by down-regulating API5 expression, thereby inhibiting E2F1 recruitment to the *Apaf-1* promoter. The expression of Apaf-1 is then blocked, and apoptosome formation is impaired. PB1-F2 interacts with ANT3 and VDAC1, resulting in increased permeability of the mitochondrial outer membrane and induction of cytochrome c release.

The viral protein PB1-F2 encoded by *PB1*, which is localized in the mitochondria of MDCK cells infected with IAV, has been shown to induce intrinsic apoptosis (type I) (Gibbs et al., 2003; Zamarin et al., 2005). *In vitro*, microinjection of PB1-F2 into MDCK cells causes its mitochondrial localization, which results in swelling and fragmentation, nuclear shrinkage, and cell death (Zamarin et al., 2005). In a previous study, the release of cytochrome c tagged with green fluorescent protein (Cc-GFP) from mitochondria was observed using flow cytometry when HeLa cells that contained a fusion protein consisting of Cc-GFP overexpressed in the mitochondria were transfected with PB1-F2. The release of Cc-GFP from mitochondria was observed in about 55% of cells in the PB1-F2 treatment group, compared with only 7% in the control group (Chen et al., 2001). In addition, when PB1-F2 was co-transfected with the anti-apoptotic protein Bcl-2 in Cc-GFP HeLa cells, release of Cc-GFP was decreased. Moreover, PB1-F2 can interact with adenine nucleotide translocase 3 (ANT3) and voltage-dependent anion-selective channel 1 (VDAC1), which are critical components of the pore complex on the mitochondrial membrane. When bongkrekic acid, a ANT3 inhibitor, was incubated with PB1-F2, apoptosis was also blocked partially (Figure 1; Zamarin et al., 2005).

In addition, the IAV nucleoprotein (NP), which is an essential part of the vRNP, also plays vital roles in promoting apoptosis and viral proliferation (Nailwal et al., 2015). NP induces apoptosis by hijacking host proteins such as clusterin precursor (CLU), RING finger protein 43 (RNF43), and apoptosis inhibitor 5 (API5). CLU was the first host protein identified, and has been shown to interact with NP to regulate apoptosis. CLU binds with Bax to inhibit the mitochondrial translocation of Bax and prevent apoptosis. However, when NP interacts with CLU, the interaction blocks the binding of CLU and Bax, thereby promoting apoptosis (Figure 1). Previous studies have evaluated the p53-Bax mitochondrial apoptosis network (McCurrach et al., 1997; Chipuk and Green, 2004; Chipuk et al., 2004; Ohtsuka et al., 2004) and shown that p53 is degraded by the host protein RNF43 through ubiquitination; however, ubiquitination is hampered when NP binds with RNF43, thereby promoting apoptosis (Figure 1; Nailwal et al., 2015). API5 is an anti-apoptotic protein whose mRNA and protein levels are down-regulated when NP is overexpressed or when the cells were infected with IAV (Mayank et al., 2015). Further studies have demonstrated that NP promotes apoptosis by down-regulating API5, thereby interfering with E2F1. This blocks recruitment of E2F1 to the apoptotic protease-activating factor-1 (*Apaf-1*) promoter, resulting in downregulation of *Apaf-1* and thereby impairing apoptosome formation (Figure 1; Mayank et al., 2015). Neuraminidase (NA) protein functions primarily to facilitate the release of progeny virions. However, a recent study showed that NA also interacts with carcinoembryonic antigen-related cell adhesion molecule 6 (CEACAM6) to increase the phosphorylation of Src, AKT, and Bcl-2, resulting in inhibition of apoptosis and promotion of cell survival (Figure 1; Gaur et al., 2012).

The microenvironmental perturbations caused by IAV infection include autophagy and ER stress, both of which are implicated in IAV-induced apoptosis (Frabutt et al., 2018; Yeganeh et al., 2018). Both hemagglutinin (HA) which is responsible for viral attachment to cell-surface sialic acid residues and subsequent fusion of the viral and host cell membranes and NA proteins trigger the ER stress response (Frabutt et al., 2018; Slaine et al., 2021). Upon IAV infection, the ER stress markers (transcription factor 6 (ATF6) and endoplasmic reticulum protein 57-kDa (ERp57) are significantly upregulated compared with that in the control group, whereas RNAi knockdown of ATF6 and ERp57 reduces the activity of caspase-3 (Roberson et al., 2012). In addition to the ER stress pathway, autophagy is also implicated in IAV-induced apoptosis. One major characteristic of autophagy is the formation of autophagosomes, which capture cellular components, and fuse with lysosomes, where the cargo is degraded (Gannage et al., 2009; Maiuri et al., 2010). Upon IAV infection, autophagosomes accumulate, and the formation of autophagosome-lysosome complexes can be blocked by M2 ion channels, which function to facilitate fusion between the viral envelope and the endosomal membrane to mediate genome uncoating; these complexes the interact with ATG5/Beclin-1, enabling evasion of autophagy-mediated viral clearance, which significantly enhances apoptosis following IAV infection (Gannage et al., 2009).

The apoptosome consists of *Apaf-1, Apaf-2* (Cyt c), *Apaf-3* (caspase-9 precursor), and dATP. When Apaf-1 interacts with Apaf-3 through its N-terminal CARD domain in the presence of Apaf-2 and dATP, pro-caspase-9 is activated and converted to effector caspase-9, which initiates the caspase cascade (Liu et al., 1996; Li et al., 1997). Furthermore, Apaf-1 interacts with the C-terminal of heat shock protein 70 (Hsp70), which inhibits apoptosome formation and reduces apoptosis; however, M1 can bind with the substrate-binding domain of Hsp70 and interferes with the anti-apoptotic function of Hsp70 (Figure 1; Halder et al., 2011).

DAI (ZBP1/DLM-1) has been shown to act a cytoplasmic DNA sensor (Wang et al., 2008; Rebsamen et al., 2009; DeFilippis et al., 2010). In addition, upon IAV infection, pattern recognition receptors (PRRs) are also involved in the mechanism of cell death. In particular, DAI, which belongs to the cytosolic DNA sensor family, is one of the most important PRRs and, directly participates in IAV-induced cell death (here, we limit our discussion to its role in apoptosis). Recent studies have demonstrated that vRNPs with defective viral genomes are directly sensed by the Zα domains of DAI (Thapa et al., 2016; Kesavardhana et al., 2017; Wang et al., 2019; Zhang T. et al., 2020).

During IAV infection, DAI recruits RIPK3 to activate downstream signaling via its RIPK homotypic interaction motif (RHIM) domains (Thapa et al., 2016; Kesavardhana et al., 2017). The activation of caspase-8 and caspase-3 is abrogated in DAI^–/–^and RIPK3^–/–^ cells, thereby inhibiting apoptosis (Kuriakose et al., 2016). Accordingly, IAV may promote apoptosis through the DAI/RIPK3/caspase-8 axis (Figure 1).

Non-structural protein 1 (NS1), which is encoded by the IAV *NS* gene, can antagonize innate immunity, e.g., by suppressing IFN-I secretion, and promote viral replication. NS1 has also been shown to be involved in the regulation of apoptosis; however, the mechanism is still unclear (Schultz-Cherry et al., 2001; Zhirnov et al., 2002; Lam et al., 2008). A comparison of apoptosis in cultured cells infected with wild-type (WT) IAVs (A/PR/8/34) and recombinant viruses with a mutant *NS1* gene (Schultz-Cherry et al., 2001) showed that typical apoptotic features, such as DNA fragmentation, were observed in WT IAVs, but not in mutant virus (Figure 1; Schultz-Cherry et al., 2001). Further studies are needed to assess the molecular mechanisms.

## Extrinsic Apoptosis

**FIGURE 2:**
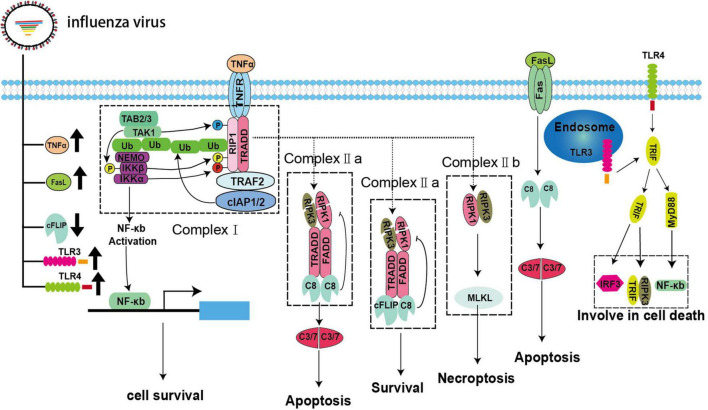
Extrinsic apoptosis and necroptosis pathways during IAV infection. IAV infection can lead to upregulation of the death receptor ligands TNFα and FasL and the pattern recognition receptors TLR3/4 as well as downregulation of cFLIP.

In addition to intrinsic pathways, IAV can mediate apoptosis via extrinsic signaling. The extrinsic apoptosis pathway is mediated by specific cell surface death receptors belonging to the tumor necrotic factor (TNF) receptor (TNFR) family, namely, TNFR1, Fas, and TRAIL-R1/2 (Figure 2; Nagata, 1999; MacFarlane et al., 2005; Vanden Berghe et al., 2015). All of these receptors possess an intracellular death domain (DD), which can recruit downstream adaptor proteins to assemble a set of complexes. The complexes activate caspase-8 and then induce apoptosis through a variety of homotypic interactions (Lee et al., 2012; Nair et al., 2014). These processes are tightly regulated and connected with necroptosis (Guo et al., 2015). TNFR1 plays a major role in mediating extrinsic apoptosis, and this process has been studied thoroughly. Here, we will use the TNRF1 pathway as an example to explain progress in studies of extrinsic apoptosis.

The binding of TNFα and TNFR1 does not always result in cell death. The fate of the cell, i.e., whether it survives or dies, is controlled by multiple key checkpoints (Vanden Berghe et al., 2015). When TNFR1 is activated, it recruits downstream adaptors, such as TRADD, TRAF2, RIPK1, and E3 ubiquitin ligases, and binds to cellular inhibitor of apoptosis 1 (cIAP) 1 and (cIAP2) to assemble complex-I via its DD (Dondelinger et al., 2013; Annibaldi and Meier, 2018). RIPK1 is subsequently ubiquitinated by cIAPs to form linear ubiquitins (Annibaldi and Meier, 2018). Then, these ubiquitin chains act as a signaling scaffold to recruit the kinase complex transforming growth factor (TGF)-β-activated kinase 1 (TAK1)/TGF-β activated kinase 1 binding protein (TAB) 2/TAB3/IκB kinase (IKK) after TNFα stimulation (Jaco et al., 2017; Annibaldi and Meier, 2018). The dissociation of RIPK1 from complex-I to assemble complex-II and its combination with the downstream adaptor protein FADD and pro-caspase-8 are dependent on some vital checkpoints (Annibaldi and Meier, 2018). In complex-I, RIPK1 is directly phosphorylated by TAK1/IKKα/IKKβ to inhibit complex-II formation and apoptosis (Dondelinger et al., 2015; Annibaldi and Meier, 2018). In addition, MK2 and nuclear factor (NF)-κB can also be activated by TAK1 and IKK, respectively, resulting in the phosphorylation of cytoplasmic RIPK1, thereby, impairing complex-II assembly and the production of pro-survival molecules, such as cellular FADD-like interleukin (IL)-1β-converting enzyme-inhibitory protein (cFLIP), to inhibit the activation of caspase-8 (Dondelinger et al., 2017; Jaco et al., 2017; Annibaldi and Meier, 2018). However, little is known about the strategies developed by IAVs to modulate extrinsic apoptosis. Fortunately, IAV infection may upregulate pro-apoptotic TNFα and FasL and downregulate anti-apoptotic cFLIP *in vivo* and *in vitro*; therefore, we speculate that IAVs can mediate apoptosis via TNFα, FasL, and cFLIP (Figure 2; Herold et al., 2008; Monteerarat et al., 2010). Additionally, influenza viruses that use other strategies to manipulate extrinsic apoptosis cannot be excluded.

## Necroptosis During Influenza a Virus Infection

Necroptosis is a newly identified mode of programmed cell death that can be triggered in multiple ways. Based on the molecular events involved, necroptosis can be divided into canonical and non-canonical pathways and is intimately associated with apoptosis through RIPK1 in the canonical pathway (Green and Llambi, 2015). In this pathway, RIPK1, TRADD, and FADD recruit different types of downstream adaptor proteins and assemble into a dynamic complex called complex-IIa after de-ubiquitination, checkpoint escape, and dissociation from complex-I. Complex-IIa determines the ultimate fate of the cell. The homodimer of pro-caspase-8 is recruited to form complex-IIa, which activates pro-caspase-8, thereby inducing apoptosis (Green and Llambi, 2015). Typically, cFLIP, which is a structural analog of pro-caspase-8, recruits complex-IIa to form heterodimers with pro-caspase-8 and prevent its activation (Green and Llambi, 2015). As shown in Figure 2, both pro-caspase-8 homodimer and pro-caspase-8/cFLIP heterodimer can cleave essential necrosis modulators, such as RIPK1 and RIPK3. Thus, pro-caspase-8/cFLIP heterodimer can inhibit both apoptosis and necroptosis (Green and Llambi, 2015). However, when the function of either caspase-8 or cFLIP is suppressed, RIPK1 and RIPK3 forms an amyloid-like, intracellular complex-IIb (necrosome) to transmit the necroptosis signal through the downstream protein MLKL. MLKL is a pseudokinase that is phosphorylated and transferred to the cellular membrane after forming oligomers. Then, a pore is formed on the cellular membrane, which can lead to leakage of intracellular contents and induce necroptosis (Green and Llambi, 2015). However, the extent to which IAV is involved in this process is unclear. To date, only NS1 has been shown to interact with MLKL and induce its oligomerization, followed by disruption of the cell membranes (Figure 1; Gaba et al., 2019). Toll-like receptor (TLR) 3/4—mediated necroptosis is another non-canonical pathway that involves the downstream adaptor TIR-domain-containing adapter-inducing IFN-β (TRIF), which contains a C-terminal RHIM motif, and recruit RIPK3 to participate in necroptosis (He et al., 2011; Tsai et al., 2014). In fact, TLR3/4 are significantly upregulated *in vivo and in vitro* during IAV infection, and MyD88 and TRIF can be recruited to activate the downstream signaling molecules IFN regulatory factor 3 and NF-κB, which are closely related to inflammation and cell death (Figure 2; Le Goffic et al., 2007; He et al., 2011; Matsumoto et al., 2011; Tsai et al., 2014, 2015; Ma et al., 2020; Bertheloot et al., 2021). Current studies have suggested that IAV may trigger necroptosis through the TLR3/4/TRIF pathway, although direct evidence for IAV-induced necroptosis through this axis is lacking (Figure 2; Le Goffic et al., 2007; He et al., 2011; Matsumoto et al., 2011; Tsai et al., 2014, 2015).

In addition to its roles in apoptosis, DAI has also been associated with necroptosis. vRNPs are sensed by DAI, which then recruits the downstream protein RIPK3 and activates MLKL to induce necroptosis (Kuriakose et al., 2016; Thapa et al., 2016; Kesavardhana et al., 2017). Necrosomes and the phosphorylated form of MLKL in DAI^–/–^ murine embryo fibroblasts or LET1 cells were undetected after IAV infection (Thapa et al., 2016). However, when MLKL^–/–^ murine bone marrow-derived macrophages (BMDMs) were infected with IAV, the activated forms of caspase-1 and caspase-8 were detected (Kuriakose et al., 2016). Furthermore, cell death in MLKL^–/–^ BMDMs showed no difference compared with that in WT BMDMs (Kuriakose et al., 2016). Intriguingly, a broad spectrum caspase inhibitor, Z-VAD-FMK, prevented cell death in MLKL^–/–^ BMDMs (Kuriakose et al., 2016). These results demonstrated that multiple cell death pathways, including necroptosis, could be activated in IAV-infected cells. Moreover, the HA protein of seasonal IAV seems to be involved in necroptosis (Hartmann et al., 2017). However, the underlying mechanism has not yet been elucidated (Table 1 and Figure 1).

## Pyroptosis During Influenza a Virus Infection

For many years, pyroptosis was regarded as a caspase-1 mediated cell death mechanism in monocytes infected with bacteria, and caspase-1 was considered the executioner of pyroptosis (Shi et al., 2017). However, caspase-1 is now considered to a downstream product of inflammasomes, including AIM2/ASC, NLRC4, NLRP3/ASC, Pyrin/ASC, and NLRP1. The real pyroptosis executioner is gasdermin D (GSDMD). GSDMD is cleaved by caspase-1, which leads to exposure of the N domain of GSDMD and pore formation, thereby inducing pyroptosis (Ding et al., 2016). In an addition to caspase-1, caspases-4, −5, −8, and −11 have been shown to cleave GSDMD and trigger pyroptosis directly via a non-canonical pathway (Shi et al., 2014, 2017; Orning et al., 2018).

**FIGURE 3:**
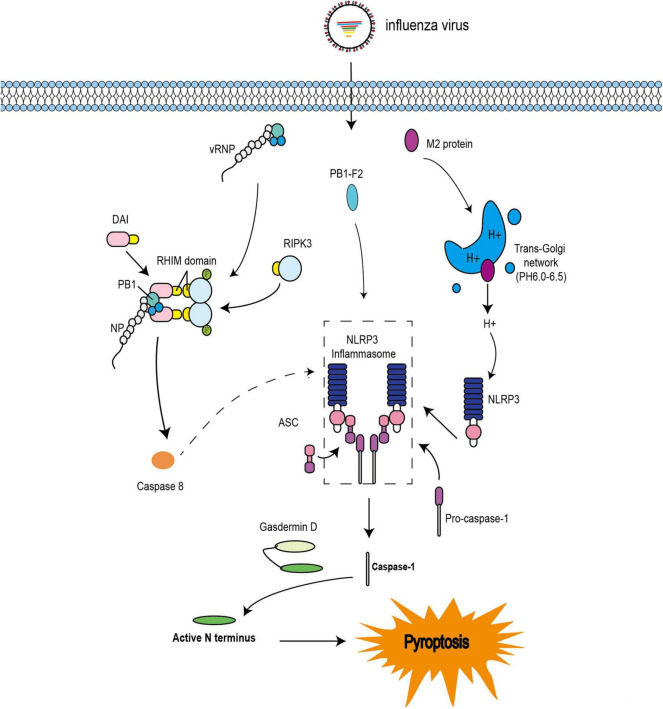
Pyroptosis pathways induced by IAV. The IAV progeny ribonucleoprotein complex (vRNP) is sensed by DAI via recruitment of RIPK3 to activate caspase-8. Caspase-8 may cleave GSDMD, and the N-terminal GSDMD fragment then activates NLRP3-dependent caspase-1. PB1-F2 can induce ASC speck formation, NLRP3 activation, and cleavage of caspase-1. M2 is translocated to the Golgi apparatus as a proton ion channel to alter the pH of intracellular compartments and activate inflammasomes.

Pyroptosis has also been observed during IAV infection. Indeed, PB1-F2 from PR8 and H7N9 viruses was shown to induce ASC speck formation, NLRP3 activation, and IL-1β secretion (Pinar et al., 2017). These results are consistent with previous studies (Figure 3; McAuley et al., 2013). The influenza virus M2 protein was found to activate inflammasome and induce pyroptosis by modifying intracellular pH (Figure 3; Ichinohe et al., 2010). Several studies have indicated that DAI is also involved in the regulation of pyroptosis during IAV infection (Kuriakose et al., 2016; Thapa et al., 2016). A previous study demonstrated that DAI recognize vRNPs and triggers pyroptosis during IAV infection. Moreover, in DAI^–/–^ or RIPK3^–/–^caspase-8^–/–^ BMDMs, the active form of caspase-1 is completely abolished. Even in RIPK3^–/–^ BMDMs, the activation of caspase-1 is extremely reduced compared with that in WT BMDMs. IL-1β and IL-18 are also significantly downregulated (Kuriakose et al., 2016). These results indicate that RIPK3-caspase-8 is located upstream of GSDMD and downstream of DAI; however, it is not clear how the DAI–RIPK3–caspase-8 signal induces pyroptosis. Nevertheless, GSDMD is cleaved by caspase-8 (Orning et al., 2018; Sarhan et al., 2018) and the N-terminal GSDMD fragment, which is the active form of GSDMD, triggers the activation of NLRP3-dependent caspase-1 via non-canonical inflammasome signaling (Kayagaki et al., 2015). Based on these studies, IAV infection may trigger pyroptosis via the RIPK1-RIPK3-caspase-8-GSDMD axis and then induce inflammation, leading to pyroptosis via the NLRP3-dependent caspase-1 activation signal (Figure 3).

Cell death is a vital part of the immune response against viral infection, and can suppress virus replication. However, excessive cell death may induce cytokine storms, thereby worsening the disease. This contradictory phenomenon has been observed in previous studies (Kuriakose et al., 2016; Thapa et al., 2016). For example, DAI is a pivotal sensor that can sense the vRNP of IAV and induce cell death (Kesavardhana et al., 2017). In a comparative analysis of WT and DAI*^–/–^* mice infected with the PR8 influenza strain, all WT mice recovered from viral infection within 15–18 days post-infection (Thapa et al., 2016). However, 80% of DAI^–/–^ mice succumbed to IAV infection. Higher lung viral titers and longer recovery times were observed in DAI^–/–^ mice compared with those in WT mice (Thapa et al., 2016). Thus, the role of DAI in IAV infection is controversial. Kuriakose et al. (2016) reported a decrease in mortality in IAV-infected DAI^–/–^ mice, although these mice had higher lung viral titers (Kuriakose et al., 2016). Moreover, the inflammatory response and epithelial damage in IAV-infected DAI^–/–^ mice are both reduced (Kuriakose et al., 2016), consistent with the findings of Thapa et al. (2016). However, the differences between these studies remain unexplained. Over all, the results of the animal experiments indicated that IAV-mediated cell death is decreased, whereas viral replication and inflammation responses are attenuated. Further studies are needed to assess whether these features are associated with a good prognosis.

## Cross-Talk Among Apoptosis, Necroptosis, and Pyroptosis

Upon infection with IAV, apoptosis, necroptosis, and pyroptosis (PANoptosis) have been observed both *in vitro* and *in vivo* (Lee et al., 2018). DAI has been found to directly recognize vRNPs and recruit an array of molecules, namely, NLRP3, RIPK1, RIPK3, caspase-8, caspase-6, and ASC, to form a multiprotein platform called PANoptosome (Christgen et al., 2020; Zheng et al., 2020). The PANoptosome initiates the cell death signal, i.e., PANoptosis. The interaction between DAI and different adaptors activates distinct forms of cell death (Kuriakose et al., 2016; Thapa et al., 2016). DAI-RIPK1-FADD-CASPASE-8 signaling pathway promotes apoptosis (Thapa et al., 2016). The DAI-RIPK3-MLKL signaling pathway induces necroptosis (Thapa et al., 2016), and the DAI-NLRP3-CASPASE-1 signaling pathway triggers pyroptosis (Kuriakose et al., 2016). Although the critical initiator, DAI, is involved in PANoptosis during IAV infection, the molecular details of the formation and dissociation of the PANoptosome remain poorly understood. Furthermore, although vRNPs are recognized by DAI, it is unclear which cell death signal is activated preferentially, and the precise spatio-temporal order and the mechanisms of different cell death signaling cascades also remain unclear. Thus, further research focusing on elucidating of these mechanisms is necessary to identify novel targets for drug development.

## Cell Death and Inflammation

Eukaryotic cells can die via several distinct biochemical pathways, which exhibit diverse physiological and morphological features (D’Arcy, 2019). Cell death plays a critical role in organism development and host defense (Barber, 2001). IAV infection commits the cell to cell death in the form of apoptosis, necroptosis, and pyroptosis (PANoptosis) (Thapa et al., 2016). Apoptotic cells preserve membrane integrity and are considered an immunologically silent form of cell death, enabling phagocytes to engulf themselves (a process called “efferocytosis”) via phosphatidylserine on the apoptotic surface (Elmore, 2007; Poon et al., 2014). By contrast, the persistence of uncleared apoptotic bodies can lead to rupture of the plasma membrane, which releases a series of damage-associated molecular patterns and triggers the inflammatory response (Poon et al., 2014; Venereau et al., 2015). Thus, the efficient clearance of IAV-infected apoptotic bodies by phagocytes is crucial to prevent further exacerbation of the inflammatory response. Phagocytosis of apoptotic bodies and IAV-infected cells by macrophages was discovered in the bronchi alveolar lavage fluid and lung tissue (Atkin-Smith et al., 2018). However, necroptosis and pyroptosis are forms of pro-inflammatory cell death, and the contents of necrotic cells released into the extracellular space elevate inflammation via multiple PRRs (Kang et al., 2014; Venereau et al., 2015). By contrast, pyroptotic cells, through persistent activation and release of the pro-inflammatory cytokines IL-1β and IL-18, ultimately exacerbate inflammation (Kuriakose et al., 2016; Lee et al., 2018). Eventually, excessive cell death within the upper and lower respiratory tracts and lung parenchyma further exacerbates inflammation, compromises the integrity of the epithelial cell barrier, and contributes to respiratory failure (Atkin-Smith et al., 2018).

## Conclusion

Although some *in vivo* animal experiments have been performed, most of the research on programmed cell death in IAV infection has been based on *in vitro* experiments, which do not reflect the true course of the IAV infection. Despite the limitations of these experiments, the data can be utilized to elucidate the relationships among cell death, viral replication, and inflammatory responses. For example, CLU and API5 can alleviate the pro-apoptotic effects of NP, and significantly reduce cell death and suppress viral replication (Morris et al., 2006; Mayank et al., 2015). By contrast, one study showed that although the host protein CEACAM6 promotes cell survival, it also enhances viral replication (Gaur et al., 2012). It is unclear why these *in vitro* cell assays have yielded paradoxical results; however, these contradictions may be related to the differences in the influenza viral strains used, infection times, and infection dosages in the testing regime. More scientifically rigorous methodologies must be developed to address this issue. Moreover, analyses of clinical cases may provide some insights. A recent study of fatal cases of IAV infection revealed diffuse alveolar damage and cell death, providing insights into the relationships between cell death and disease prognosis (De Jong et al., 2006). Importantly, cytokine storm has also been shown to be closely associated with cell death. In addition to cytokine storm in IAV infection, this phenomenon has also been observed in severe acute respiratory syndrome coronavirus (SARS-CoV), Middle East respiratory syndrome coronavirus (MERS-CoV), and SARS-CoV-2 infections and has been shown to be associated with disease severity and mortality (Cao, 2020; Felsenstein et al., 2020; Zhang Y. et al., 2020). Accordingly, these findings suggest that excessive cell death may cause serious severe tissue damage and hypercytokinemia. Further studies are needed to investigate the relationships among cell death, inflammatory reactions, viral replication, and disease prognosis in IAV infection.

## Author Contributions

RG wrote the initial draft of the manuscript and prepared the figures. QC carefully revised and corrected the manuscript. Both authors contributed to the article and approved the submitted version.

## Conflict of Interest

The authors declare that the research was conducted in the absence of any commercial or financial relationships that could be construed as a potential conflict of interest.

## Publisher’s Note

All claims expressed in this article are solely those of the authors and do not necessarily represent those of their affiliated organizations, or those of the publisher, the editors and the reviewers. Any product that may be evaluated in this article, or claim that may be made by its manufacturer, is not guaranteed or endorsed by the publisher.
